# Environmental and occupational respiratory diseases – 1062. The climate and aero allergens in Mediterranean region and allergen sensitivity in allergic rhinoconjuctivitis and allergic asthma patients

**DOI:** 10.1186/1939-4551-6-S1-P60

**Published:** 2013-04-23

**Authors:** Arzu Didem Yalcin, Saime Basaran, Atil Bisgin, Hasan H Polat

**Affiliations:** 1 Internal Medicine, Allergy and Immunology, Education and Research Hospital, Turkey; 2 Southwest Anatolia Forest Research Institute, Antalya, Turkey, Turkey; 3 Cancer Institute, Sweden; 4 Public Health Department, Faculty of Medicine, Cumhuriyet University, Turkey

## Background

In this study, we evaluated the profiles of allergic rhino-conjunctivitis and asthma patients annually in a mediterranean coastal city Antalya in Turkey.

## Methods

As well as evaluating patients’ allergic clinical status, we also recorded the climate and pollens in the air of city center if any correlation between pollination, climatic conditions and allergic disorders. Total and specific IgE levels and skin prick tests (SPT) were evaluated. The meteorological conditions and the pollen count/cm^2^ in each month of whole year and the concordance of this with the patient’s clinical status were evaluated.

## Results

The major causes of allergic rhinoconjunctivitis symptoms were air pollens and house dust. SPT positivity for corylusavellana was significant in the age group of >40 years old. SPT positivity for plantagolanceolata, aspergillus fumigatus and d. pteronyssinus was significant in patients younger than 40 years old. Pollen count per cm2 was recorded as 1447.9 over the whole year with a maximum in May and minimum in January. The monthly pollen count for the city during a one year period. Pollens of Giraminae plant known to be very allergic were highly detected between May and November. Pollination levels are consistent from March 2010 to February 2011 and in Antalya high levels occur mostly from April to June. Thus skin prick test were performed mostly in May/June (~30%). During these months, the meteorological conditions of the city were windy with low humidity without rain and lukewarm temperatures, all of which contribute to high risk conditions for seasonal allergies.

## Conclusions

The major allergen between April and June was derived from Graminae; between February and March was Cupressus sp; and between March and June was Pinus sp. These results suggest that the pollination is correlated with allergic conditions and thus SPT might be best performed according to the pollen population.

